# Regulatory influence of α-Pinene on MATN3 expression in hepatocellular carcinoma: Extending to pan-cancer analysis

**DOI:** 10.1371/journal.pone.0330653

**Published:** 2025-09-26

**Authors:** Binyu Zhao, Wenliang Qiu, Wenhui Xu, Lixin Wu, Jiayin Zhao, Haocheng Zhao, Fengxiang Wei, Bing Qiu

**Affiliations:** 1 Jiamusi University, Jiamusi City, Heilongjiang Province, China; 2 Department of Gastroenterology, Heilongjiang Provincial Hospital, Harbin City, Heilongjiang Province, China; 3 Mudanjiang Medical University, Mudanjiang City, Heilongjiang Province, China; 4 The Genetics Laboratory, Longgang District, Maternity & Child Healthcare Hospital of Shenzhen, Guangdong Province, China; The University of Haripur, PAKISTAN

## Abstract

In the search for novel therapeutic strategies against cancer, natural compounds such as α-Pinene, a bioactive monoterpene predominantly found in coniferous trees, have emerged as potential modulators of oncogenic processes. The primary objective of this study was to investigate whether α-Pinene can regulate the expression of MATN3 (Matrilin-3), a gene previously associated with poor prognosis in various cancers. Our central hypothesis is that α-Pinene exerts its anticancer effects, at least in part, by downregulating MATN3, thereby impacting oncogenic signaling. The rationale for selecting MATN3 as a target lies in its demonstrated overexpression in multiple cancer types and its established role as both a prognostic marker and a contributor to tumor progression. Extending our research through a comprehensive pan-cancer analysis, we confirmed the association between high MATN3 expression and poor prognosis across multiple cancer types. Further exploration revealed a strong correlation between MATN3 expression and immune infiltration, as well as significant associations with key immunomodulatory genes, suggesting an intricate role of MATN3 in the tumor immune environment. Enrichment analysis pointed to the involvement of MATN3 in the PI3K-AKT signaling pathway. Additionally, we identified connections between MATN3 and extracellular matrix (ECM)-related mechanisms.The downregulation of MATN3 by α-Pinene in HepG2 cells suggests a novel mechanism by which α-Pinene may exert anticancer effects, potentially through the disruption of key oncogenic and immune-related pathways. This study not only underscores the anticancer potential of α-Pinene but also establishes MATN3 as a critical target and biomarker across a spectrum of cancers. Our findings advocate for further clinical investigations into α-Pinene’s therapeutic potential, particularly focusing on its impact on MATN3 and related pathways in diverse cancer contexts.

## Introduction

In the relentless pursuit of innovative cancer therapeutics, the exploration of natural compounds has unveiled promising avenues for intervention [1,2]. Among these, α-Pinene, a bioactive monoterpene predominantly derived from coniferous trees, has garnered attention for its potential regulatory effects on key oncogenic processes [3–6]. Our research team previously investigated the anticancer effects of pine needle oil and found that it induces G2/M cell cycle arrest in HepG2 cells, with α-Pinene identified as a key active component [7]. In non-small cell lung cancer, gastric cancer, melanoma, hepatocellular carcinoma and prostate cancer, α-Pinene has demonstrated potential anticancer properties [6,8–12]. Furthermore, α-Pinene may also play a role in the treatment of hematological malignancies, as a study indicates that it possesses anticancer potential in T-cell tumors [13]. Our study delves into the mechanistic interactions between α-Pinene and cancer biology, with a specific focus on its impact on gene expression within HepG2 cells, a well-established hepatocellular carcinoma model. In this study, the selection of MATN3 as a target was guided by our RNA sequencing data, which identified MATN3 as one of the most significantly downregulated genes after α-Pinene treatment in hepatocellular carcinoma cell line. This data-driven discovery, combined with MATN3’s known oncogenic role and involvement in the extracellular matrix and PI3K-AKT pathway, led us to hypothesize a novel regulatory link between α-Pinene and MATN3. We found the regulatory relationship between α-Pinene and MATN3 expression has not yet been reported in the literature. Therefore, our findings may provide new insights into the potential mechanisms by which α-Pinene could exert anticancer effects, particularly through the modulation of MATN3 and its associated pathways.

MATN3 is correlated with patient survival in several cancers, particularly in gastric cancer, where it may play a pivotal role. However, the potential functions of MATN3 have not been discovered in a broader array of tumors [14–18]. Its role transcends that of a mere prognostic marker, extending to significant participation in oncogenic signaling pathways [19]. Our comprehensive pan-cancer analysis further corroborates the link between elevated MATN3 expression and poor prognosis across multiple cancer types, emphasizing its potential as both a therapeutic target and a diagnostic biomarker.

## Method

### Data acquisition

We obtained the expression data for MATN3 in both cancerous and normal tissues from the TCGA (The Cancer Genome Atlas) and GTEx (Genotype-Tissue Expression) datasets through the XENA database (https://xenabrowser.net/) [20]. These datasets had already undergone batch effect correction and log2(X + 1) transformation by the XENA database, allowing for direct comparison. Additionally, we downloaded data from the Cancer Cell Line Encyclopedia (CCLE) database (https://sites.broadinstitute.org/ccle/) [21], extracting MATN3 expression levels and annotative information on tumor cell lines to facilitate comparative analysis across different cell line groups. The mutation and copy number variation (CNV) data for tumors were sourced from cBioPortal (https://www.cbioportal.org/). TCGA: Data were obtained via UCSC Xena (GDC TCGA hub; “TCGA Pan-Cancer (PANCAN) gene expression RNA-seq” HTSeq counts/FPKM/TPM) and the GDC Data Portal; DOI 10.1038/ng.2764; no single accession (https://xenabrowser.net/datapages/?cohort=TCGA%20Pan-Cancer%20(PANCAN)).

GTEx: We used GTEx RNA-seq gene expression from the GTEx Portal and UCSC Xena (GTEx hub; “GTEx RNA-seq”); DOI 10.1126/science.aaz1776; no accession number (https://xenabrowser.net/datapages/?cohort=TCGA%20TARGET%20GTEx).

CCLE: RNA-seq expression from the DepMap/CCLE resource with accession PRJNA523380 and DOI 10.1038/s41586-019-1186-3; retrieval via DepMap → Data → Downloads → “CCLE_expression” with matching annotations (https://sites.broadinstitute.org/ccle).

cBioPortal: Portal used to access harmonized matrices and clinical/alteration summaries; no accessions or DOIs (https://www.cbioportal.org).

OMIX (this study): RNA-seq count data from HepG2 after 24 h α-Pinene (50 μM) treatment are deposited under OMIX011684; file “OMIX011684-01 Count Data”; BioProject PRJCA042366; no DOI (https://ngdc.cncb.ac.cn/omix/release/OMIX011684).

### Survival analysis

We downloaded curated clinical data from the XENA database, which includes survival information for all TCGA patients [20,22]. Patients were grouped based on the median expression of MATN3, and inter-group survival differences were analyzed using the “survival” R package. Visualization was performed using the “survminer” and “ggplot2” packages. Furthermore, we constructed a Cox proportional hazards regression model to assess the relationship between MATN3 expression and survival outcomes across various cancers, with the results visualized using the “forestplot” R package.

### Tumor heterogeneity

Drawing upon previous literature [23], we obtained data on tumor purity [24], microsatellite instability (MSI) [25], homologous recombination deficiency (HRD) [24], and tumor mutational burden (TMB) for each sample [26]. We then calculated the correlations between MATN3 expression and these tumor heterogeneity metrics.

### Immune infiltration and immune genes

We employed three distinct algorithms—EPIC, ESTIMATE, and TIMER—to calculate immune cell infiltration scores for each sample [27–31]. Following the computation of these scores, we conducted correlation analyses.

For immune-related genes, we referenced previous studies to obtain corresponding gene expression matrices from the XENA database [23,32,33]. These matrices included immune checkpoint inhibitory and stimulatory genes, as well as chemokine, immuno-inhibitor, and immuno-stimulator genes. We then merged these with MATN3 expression data and calculated the correlations. The results were visualized using heatmaps.

### Protein interaction network

Using the STRING (Search Tool for the Retrieval of Interacting Genes/Proteins) database (https://cn.string-db.org/), we retrieved proteins associated with MATN3 in humans [34–37]. Subsequently, we employed the “igraph” package to visualize the interaction network. Ultimately, we identified 41 proteins related to MATN3.

### Enrichment and differential analysis

We conducted enrichment analysis using the “clusterProfiler” package [38], with annotations provided by the “org.Hs.e.g.,db” package. For differential analysis of the obtained transcriptomic data, we utilized the “limma” package [39]. In each cancer type, we grouped samples based on the median expression of MATN3 and performed differential expression analysis between high and low expression groups to identify differentially expressed genes. Subsequently, we used “clusterProfiler” to perform Gene Set Enrichment Analysis (GSEA) to ascertain pathways associated with differential MATN3 expression in each cancer type. For Protein-Protein Interaction (PPI) analysis, we performed KEGG (Kyoto Encyclopedia of Genes and Genomes) and GO (Gene Ontology) enrichment analyses.

### Cell culture

Two hepatocellular carcinoma (HCC) cell lines, HepG2 and Hep3B, were employed in this study. The cells were procured from MeisenCTCC. Both cell lines were cultured in Eagle’s Minimum Essential Medium (EMEM) (MeisenCTCC, Cat. CTCC-009–011) supplemented with 10% fetal bovine serum (FBS) (GIBCO, Cat. 10099141C) and 1% Penicillin-Streptomycin (Invitrogen, Cat. 15140−122). The cell cultures were maintained at 37°C in an environment containing 5% carbon dioxide.

### Cell proliferation assay

We conducted cell proliferation assays using the CCK8 (Cell Counting Kit-8) kit (Biosharp, Cat. BS350B CCK8). The cells were treated with varying concentrations of α-Pinene (MCE, Cat. HY-N0549), and post-treatment cell viability was compared with the control group. Cells were treated with α-Pinene at concentrations of 0, 25, 50, and 100 μM. These concentrations were selected based on preliminary dose-response studies [7,10,12]. The CCK8 assay was performed according to the manufacturer’s instructions.

### RNA extraction and RT-qPCR

RNA was extracted using Trizol reagent (Thermo Fisher, Cat. 15596026). Reverse transcription was performed with the Go Script Reverse Transcription System (Promega, Cat. A5000-1). Quantitative PCR (qPCR) was carried out using the GoTaq qPCR Master Mix (Promega, Cat. A6001). All procedures were conducted according to the manufacturers’ protocols.

The primer sequences for MATN3 (5’-3’) are as follows:
Forward Primer: TCTCCCGGATAATCGACACTC
Reverse Primer: CAAGGGTGTGATTCGACCCA
The primer sequences for GAPDH (5’-3’) are as follows:
Forward Primer: GTCTCCTCTGACTTCAACAGCG
Reverse Primer: ACCACCCTGTTGCTGTAGCCAA

### Western blot

Cells were treated with DMSO (Dimethyl Sulfoxide) as control, 50 μM α-Pinene, or 100 μM α-Pinene for 24 hours. Total protein was extracted using RIPA lysis buffer supplemented with protease and phosphatase inhibitors (Beyotime, China). Protein concentrations were determined by BCA protein assay kit (Beyotime, China), and equal amounts of protein were separated by SDS-PAGE and transferred to PVDF membranes (Millipore, USA). Membranes were blocked with 10% non-fat milk in TBST for 1 hour at room temperature and then incubated overnight at 4°C with the following primary antibodies: PI3 Kinase p85 (CST#4292), Phospho-PI3 Kinase p85 (CST#4228), AKT (abclonal#A18675), Phospho-AKT (abclonal#AP0637), MATN3 (abclonal#A7700), GAPDH (abclonal#AC002, loading control). After washing, membranes were incubated with HRP-conjugated secondary antibodies for 1 hour at room temperature. Protein bands were visualized using enhanced chemiluminescence reagents.

### RNA-sequencing

α-Pinene was added to normally cultured HepG2 cells to a final concentration of 50 μM. After 24 hours of treatment, RNA was extracted from both the treated and control groups. The extracted RNA was then sent to Novogene (https://cn.novogene.com/) for RNA sequencing. RNA integrity was assessed using the Agilent 2100 Bioanalyzer, and only samples with an RNA integrity number (RIN) greater than 8.0 were used for library construction. For each sample, 3 μg of total RNA was used to deplete ribosomal RNA and construct strand-specific cDNA libraries following the manufacturer’s protocol. Sequencing libraries were prepared using the NEBNext Ultra Directional RNA Library Prep Kit. Sequencing was performed on an Illumina HiSeq 4000 platform, generating paired-end reads. Raw sequencing data quality was assessed using FastQC. The quality control criteria required that the percentage of bases with a Phred quality score above 20 (Q20) exceeded 90%, and the percentage above 30 (Q30) exceeded 85%. Only data meeting these quality thresholds were included in downstream analyses.

### Ethical statement

No human or animal samples were involved in this study. Therefore, ethical approval was not required.

### Statistical analysis

All experiments were performed in triplicate using independent replicates (n = 3). Data are presented as mean ± standard deviation (SD). Statistical analyses were conducted using GraphPad Prism. For comparisons between two groups, an unpaired Student’s t-test was applied. For more than two groups, the ANOVA test was applied. P-value less than 0.05 was considered statistically significant.

## Result

### α-Pinene Inhibits HCC Cell Growth and Reduces MATN3 Expression

**Fig 1:**
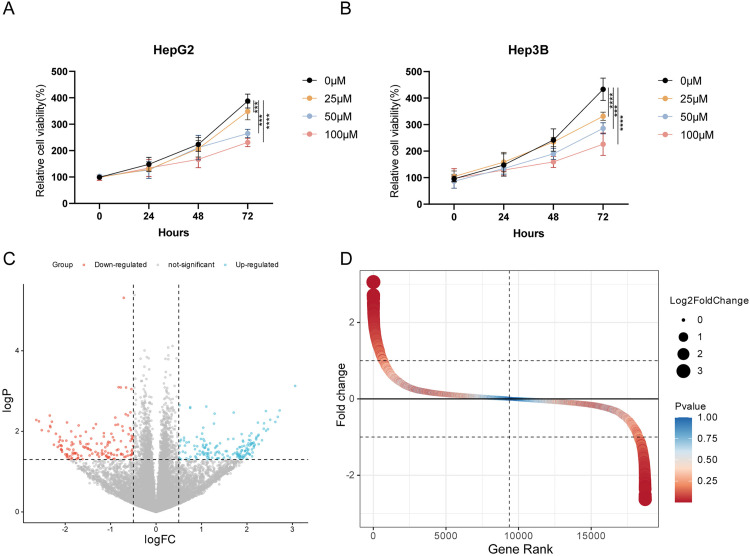
The impact of α-Pinene on hepatocellular carcinoma cell lines. (A-B) α-Pinene inhibit HepG2 and Hep3B cell proliferation. **(C)** Volcano plot illustrating the differential gene expression in HepG2 cells following treatment with α-Pinene. **(D)** Rank plot of gene expression changes in Hep3B cells treated with α-Pinene. Statistical significance: *** p < 0.001; **** p < 0.0001.

In our investigation into the anticancer effects of α-Pinene, we initially assessed its impact on the proliferation of hepatocellular carcinoma cell lines, HepG2 and Hep3B. Employing CCK8 cell viability assay, we established that α-Pinene significantly inhibited cell growth in a dose-dependent manner (Fig 1A–B). The inhibitory effects were particularly pronounced at 48- and 72-hours post-treatment, indicating a potent cytostatic effect of α-Pinene on these cancer cells.

Subsequent RNA sequencing of α-Pinene-treated HepG2 and Hep3B cells revealed a notable downregulation of MATN3 (Fig 1C–D), a gene previously associated with poor prognosis in various cancers. To validate these sequencing results, we conducted quantitative real-time PCR (qPCR) analyses. The qPCR data corroborated the RNA-seq findings, confirming a significant reduction in MATN3 mRNA levels in both cell lines following treatment with α-Pinene (Supplementary Fig 1 A-B in S1 File).

These results not only reinforce the potential of α-Pinene as an antiproliferative agent against HCC but also highlight its ability to modulate gene expression, specifically reducing levels of MATN3, which is implicated in tumor cell survival and immune escape.

### Aberrant Expression of MATN3 Across Various Cancers

Building on the findings that α-Pinene effectively reduces MATN3 expression in HCC cell lines, we expanded our investigation to explore the expression patterns and potential roles of MATN3 across a broader spectrum of cancers. This transition was prompted by the need to understand whether the observed downregulation of MATN3 could be linked to general mechanisms of tumorigenesis and tumor progression in diverse cancer types.

**Fig 2:**
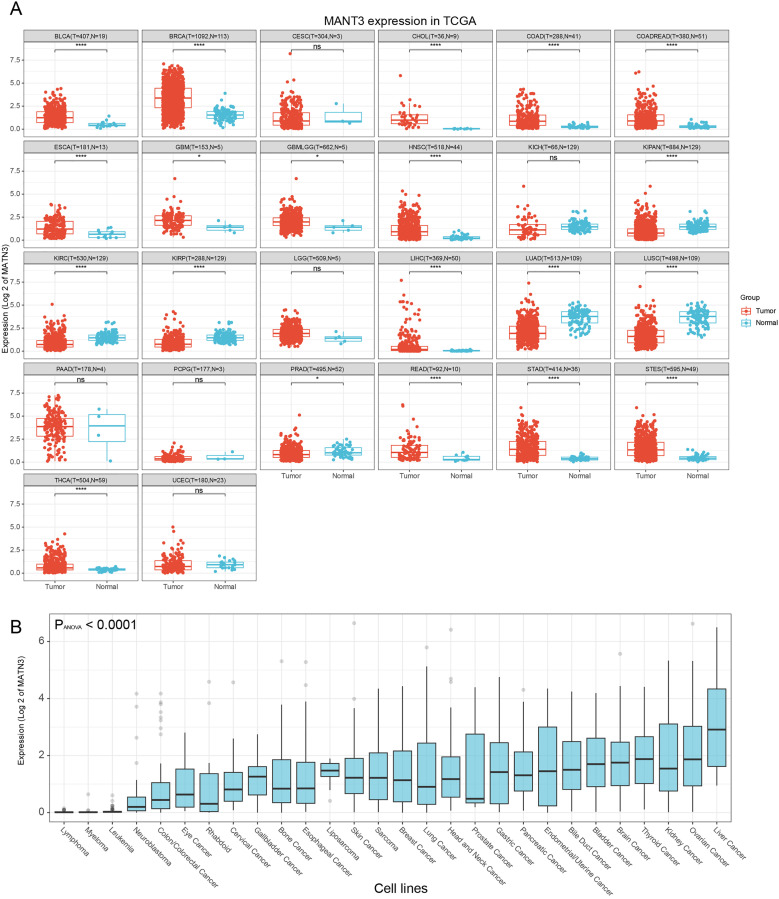
Differential Expression of MATN3 in Cancerous and Normal Tissues, and Across Cancer Cell Lines. **(A)** Expression of MATN3 in normal and tumor tissues across multiple cancer types sourced from The Cancer Genome Atlas (TCGA). **(B)** Expression levels of MATN3 across various cancer cell lines from the Cancer Cell Line Encyclopedia (CCLE).

Utilizing publicly available cancer genomic databases, we conducted a comprehensive analysis of MATN3 expression across multiple cancer cohorts. In our pan-cancer analysis, utilizing extensive cancer genomic and transcriptomic datasets, we observed a notable dichotomy in MATN3 expression. Our analysis revealed that MATN3 is consistently overexpressed in several malignancies when compared to corresponding normal tissues (Fig 2A and Supplementary Fig 2 in S1 File). This ubiquitous upregulation of MATN3 was particularly evident in cancers such as ACC, BLCA, BRCA, COAD, ESCA, GBM, LGG, HNSC, LIHC, PAAD, READ, STAD and TGCT, suggesting a common oncogenic role. However, MATN3 conversely exhibited lower expression levels in certain cancers such as KICH, KIRP, and LUSC. This pattern suggests a complex role of MATN3, potentially functioning in a context-dependent manner.

The reduced expression of MATN3 in specific cancer types like KICH, KIRP, and LUSC raises intriguing questions about its biological function and its potential as a therapeutic target. It suggests that MATN3 may play divergent roles in different tissue environments, possibly related to distinct regulatory networks or the tumor microenvironment. This complexity underscores the necessity of a nuanced approach to targeting MATN3 in cancer therapy, considering the variable expression and potential contrasting effects across different cancer types. Our findings highlight the importance of context and cancer specificity in the development of targeted therapies and further validate the relevance of exploring the mechanisms underlying MATN3 expression modulation by agents like α-Pinene.

To further elucidate the expression patterns of MATN3 in various cancers, we utilized the Cancer Cell Line Encyclopedia to analyze MATN3 expression across a wide array of cancer cell lines. As illustrated in Fig 2B, our analysis revealed a striking variation in MATN3 expression among different cancer types at the cellular level. Notably, MATN3 expression was almost undetectable in hematological malignancies’ cell lines, Conversely, liver cancer cell lines exhibited the highest levels of MATN3 expression. This observation is in alignment with our initial findings from the α-Pinene treatment studies in HCC cell lines.

### Survival analysis based on MATN3 expression

**Fig 3:**
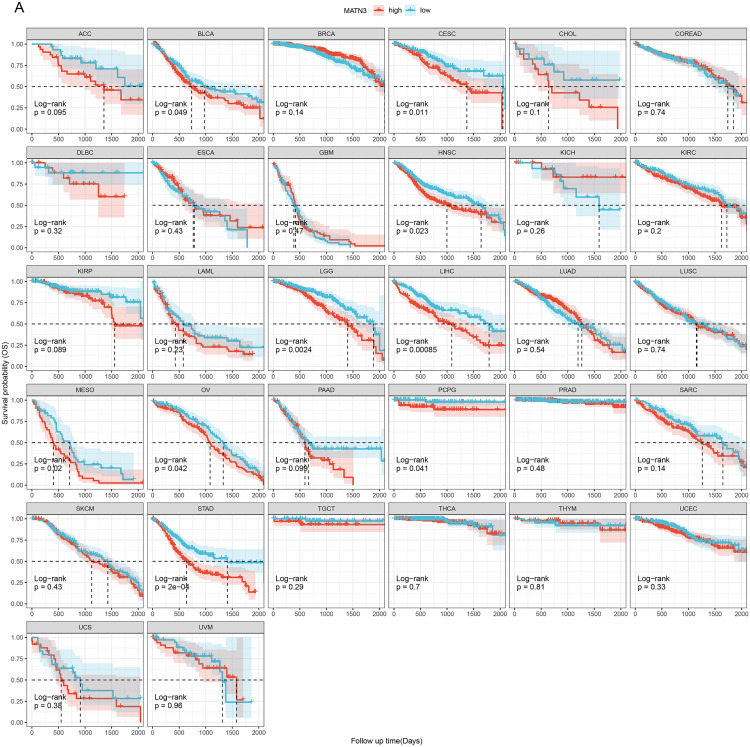
Survival Analysis of High vs. Low MATN3 Expression Across Various Cancer Types. **(A)** Kaplan-Meier survival curves comparing the overall survival between high (red) and low (blue) MATN3 expression groups across multiple cancer types. Statistical significance: * p < 0.05; ** p < 0.01; *** p < 0.001; **** p < 0.0001; ns, not significant.

Continuing our comprehensive analysis of MATN3, we examined its association with patient survival outcomes across multiple cancer types. This step was crucial to understanding the clinical significance of MATN3 expression levels in cancer prognosis. We conducted Kaplan-Meier survival analyses to compare the prognosis between high and low MATN3 expression groups across various cancers. As presented in Fig 3A, the results demonstrated that higher expression of MATN3 was significantly associated with shorter survival in patients with BLCA, CESC, HNSC, LGG, LIHC, MESO, OV, PCPG, and STAD. Logrank survival analysis revealed that high MATN3 expression was significantly associated with poorer overall survival in multiple cancer types (Supplementary Table 1 in S1 File).

To further validate the prognostic significance of MATN3, we employed Cox proportional hazards regression analysis, as depicted in Supplementary Figure 3A-B in S1 File. This analysis confirmed that high MATN3 expression served as a predictor of worse disease-specific survival (DSS) and progression-free interval (PFI) in several cancers, including STAD, GBMLGG, COAD, CESC, LIHC, ACC, PCPG, and MESO. These results indicate that MATN3 not only plays a role in survival outcomes but also significantly impacts survival outcomes, making it a valuable marker for assessing prognosis.

### MATN3 mutational landscape and tumor heterogeneity

**Fig 4:**
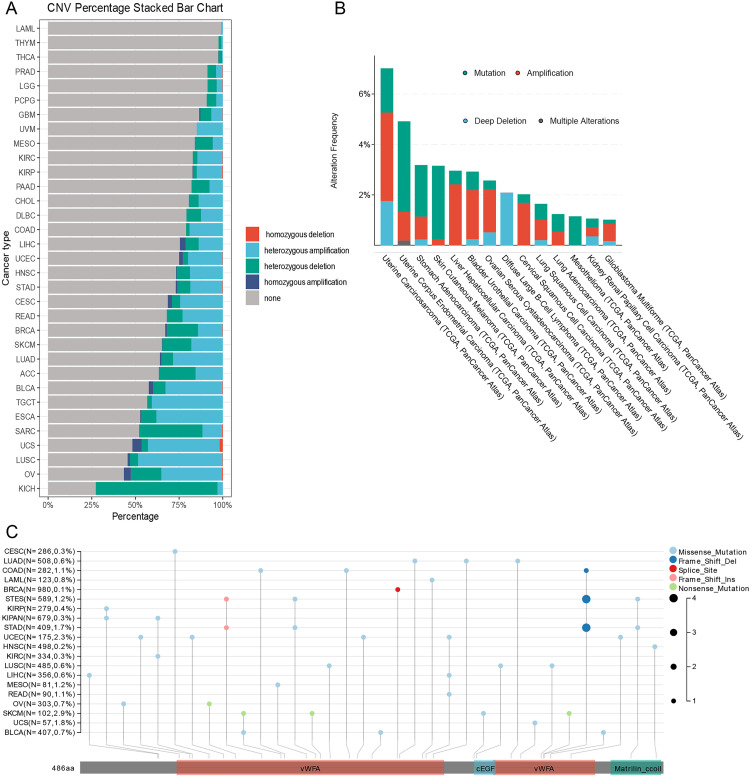
Pan-cancer analysis of Mutation and Copy Number Variation (CNV) Analysis of MATN3. **(A)** CNV percentage stacked bar chart depicting the distribution of different types of copy number variations for MATN3 across various cancer types. **(B)** Alteration frequency chart of MATN3 across different cancer types through cBioportal. **(C)** Lollipop plot illustrating the distribution and types of mutations in the MATN3 gene.

Our comprehensive examination of MATN3 has extended into the study of its mutational landscape across multiple cancer types, offering insights into its genetic alterations and the implications for tumor behavior and therapy response. The stacked bar chart (Fig 4A) illustrates the frequency of copy number variations (CNVs) affecting the MATN3 gene in various cancers. Cancers such as ovarian cancer and kidney chromophobe exhibit significant rates of homozygous deletions, which might contribute to lower MATN3 expression levels observed in these cancers, as previously discussed. In contrast, other cancers like bladder cancer and liver hepatocellular carcinoma show instances of heterozygous amplification, potentially correlating with the higher expression levels and the poorer prognosis associated with MATN3 in these conditions. Fig 4B presents a more detailed view of MATN3 mutations and amplifications across various cancers, utilizing data from the cBioPortal. The scatter plot in Fig 4C maps specific mutations within the MATN3 protein, highlighting the types of mutations and their locations within the protein structure. This visualization underscores the critical regions of the MATN3 protein that are frequently targeted by mutations, such as the von Willebrand factor A (vWFA) domains, which are crucial for protein-protein interactions [40]. Despite the diverse types of alterations observed across different cancers, the overall mutation frequency of MATN3 remains relatively low, with the wild-type allele being predominant in most cases.

The comprehensive analysis of MATN3 mutations and their correlation with various indicators of tumor heterogeneity offers insights into the complex role this gene may play across different cancer types. In the study of tumor purity, MATN3 expression shows a varying relationship depending on the cancer type (Supplementary Fig 4A in S1 File). MATN3 expression exhibits a significant negative correlation with tumor purity in several types of cancer, including PCPG, CHOL, BLCA, COAD, READ, and STAD. Conversely, in ACC, SKCM, TGCT, and THYM, MATN3 expression is significantly positively correlated with tumor purity. Tumor Mutational Burden (TMB) is a crucial metric for assessing tumor treatment. We found that MATN3 does not exhibit a significant correlation with TMB in most tumors, but it shows a negative correlation in STAD, HNSC, LUAD, and BRCA (Supplementary Fig 4B in S1 File). Microsatellite instability (MSI) is similarly used to guide chemotherapy plans for cancer patients. Our analysis revealed that MATN3 is significantly negatively correlated with MSI in STAD, COAD, LUAD, and LUSC, while it is positively correlated in TGCT (Supplementary Fig 4C in S1 File). Homologous Recombination Deficiency (HRD) is an important metric for evaluating DNA repair capability. By calculating the correlation between HRD scores and MATN3, we found that MATN3 is significantly negatively correlated with HRD in BRCA, LUSC, and LUAD. Conversely, it shows a significant positive correlation in COAD, HNSC, STAD, ESCA, PRAD, and ACC. Interestingly, in these cancers, higher MATN3 expression is associated with poorer prognosis (Supplementary Fig 4D in S1 File).

### MATN3 and the tumor microenvironment

**Fig 5:**
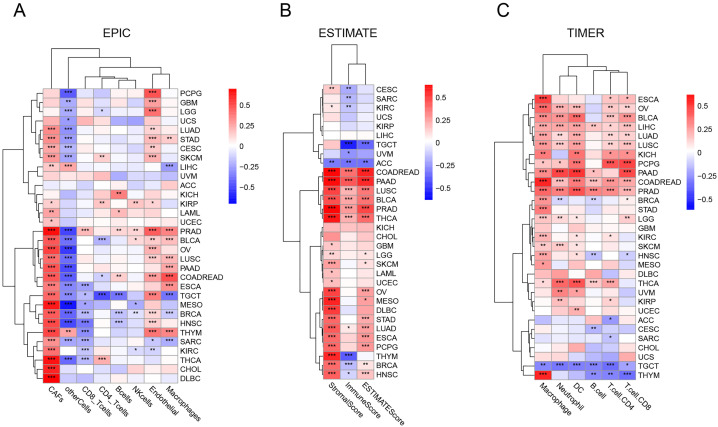
Correlation of MATN3 Expression with Immune Infiltration. Heatmap depicting the correlation between MATN3 expression and immune cell infiltration as analyzed by the **(A)** EPIC, **(B)** ESTIMATE and **(C)** TIMER across various cancer types.

After establishing a connection between MATN3 mutations and tumor heterogeneity, further analysis was conducted to examine the relationship between MATN3 and tumor immune infiltration across different cancer types using algorithms such as EPIC, ESTIMATE, and TIMER. These tools assess the levels of various immune cells within the tumor microenvironment, providing a broader understanding of how MATN3 might influence or be influenced by immune dynamics. Previous results have established a correlation between MATN3 and tumor purity, which is influenced by immune cell infiltration and the tumor microenvironment. Therefore, we further analyzed the relationship between MATN3 and immune cells. As shown in Fig 5A, the correlation between MATN3 and various tumor microenvironment cells was calculated using the EPIC algorithm. We found that cancer-associated fibroblasts (CAFs) exhibit a strong positive correlation with MATN3, similar to endothelial cells. The ESTIMATE algorithm yielded analogous results, showing a significant positive correlation between the stromal score and MATN3 (Fig 5B). Finally, using the TIMER algorithm, we calculated the correlation between MATN3 and several immune cells. As depicted in Fig 5C, macrophages positively correlate with MATN3, while in TGCT, MATN3 shows a negative correlation trend with various immune cells.

### MATN3 and immune regulatory genes

Following the elucidation of the relationship between MATN3 expression and immune cell infiltration across various cancer types, our research further explored the correlation between MATN3 and key immune regulatory genes. This investigation utilized extensive bioinformatics analyses to assess the expression levels of MATN3 alongside a panel of immune inhibitors, immunostimulators, checkpoint inhibitory molecules, checkpoint stimulatory molecules, and chemokines, thus providing a comprehensive insight into the immunological landscape modulated by MATN3.

**Fig 6:**
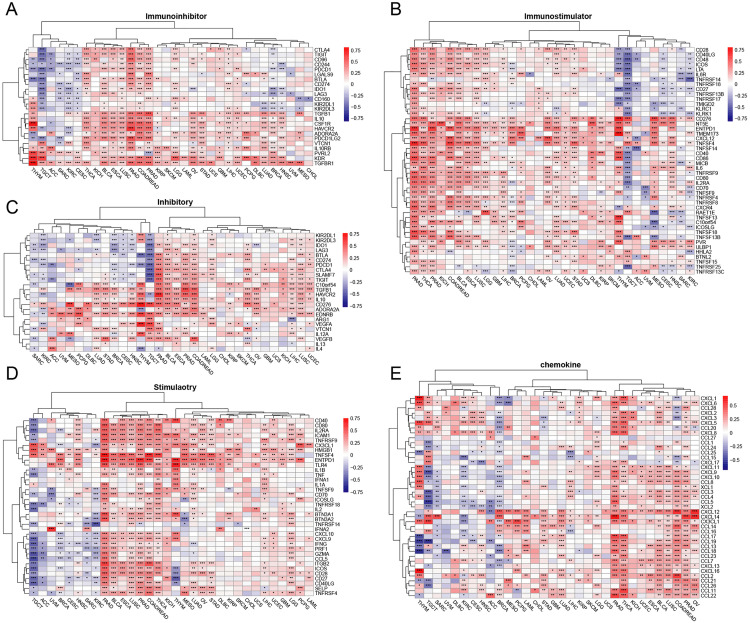
Correlation of MATN3 Expression with Immune Modulatory Factors. Heatmap showing the correlation coefficients between MATN3 expression and a range of (A) immunoinhibitory, (B) immunostimulatory, (C) inhibitory immune checkpoints, (D) stimulatory immune checkpoints and (E) chemokine across multiple cancer types.

Our findings reveal a complex interaction pattern. Figs 6A and 6B illustrate the correlation between MATN3 and immunosuppressive and immunostimulatory genes across various cancers. We observed that TGFBR1 exhibits a significant correlation with MATN3. In PAAD, PRAD, COADREAD, and LICH, MATN3 shows a positive correlation with multiple immunosuppressive genes. Interestingly, MATN3 also demonstrates a positive correlation with various immunostimulatory genes. This trend is particularly evident in PAAD, THCA, PRAD, and LICH. Additionally, MATN3 tends to positively correlate with genes such as CD276, NT5E, and TMEM173 in most cancers.

Immune checkpoints are critical regulators of the immune system, designed to maintain self-tolerance and modulate the duration and amplitude of physiological immune responses. In the realm of oncology, these checkpoints often become pivotal players in cancer pathogenesis by enabling tumor cells to evade immune surveillance [41–43]. Figs 6C and 6D display the correlation between MATN3 and immune checkpoint inhibitory and stimulatory genes across multiple cancer types. In PAAD, BLCA, ESCA, PRAD, and COADREAD, MATN3 shows a positive correlation with several inhibitory checkpoint genes (Fig 6C). CD276 and EDNRB are significantly positively correlated with MATN3 in various cancers. In contrast, in KIRC and TGCT, MATN3 tends to negatively correlate with several stimulatory checkpoint factors. Conversely, in PAAD, BLCA, ESCA, LUSC, PRAD, and COADREAD, MATN3 exhibits a positive correlation with these stimulatory factors (Fig 6D). Notably, TNFSF4 and ENTPD1 are positively correlated with MATN3 across most cancers.

Chemokines regulate the recruitment and infiltration of various immune cells into the tumor stroma, thereby modulating the immune response to neoplastic cells [44,45]. To further explore the relationship between MATN3 and chemokines in cancer, we conducted an analysis as shown in Fig 6E. We found that in PAAD, THCA, BLCA, LUSC, COADREAD, and PRAD, MATN3 exhibits a significant positive correlation with chemokines. Conversely, in ACC, BRCA, and TGCT, the correlation tends to be predominantly negative. Notably, CXCL12 shows a positive correlation with MATN3 in most cancers, suggesting a potential link between MATN3 and CXCL12.

### MATN3 enrichment analysis

To further elucidate the potential roles of MATN3 in cancer, an enrichment analysis was conducted on the gene. Initially, MATN3 expression was subjected to differential analysis based on median expression grouping. Subsequently, enrichment analyses using both KEGG pathways and Gene Set Enrichment Analysis (GSEA) were performed on the upregulated differential genes.

**Fig 7:**
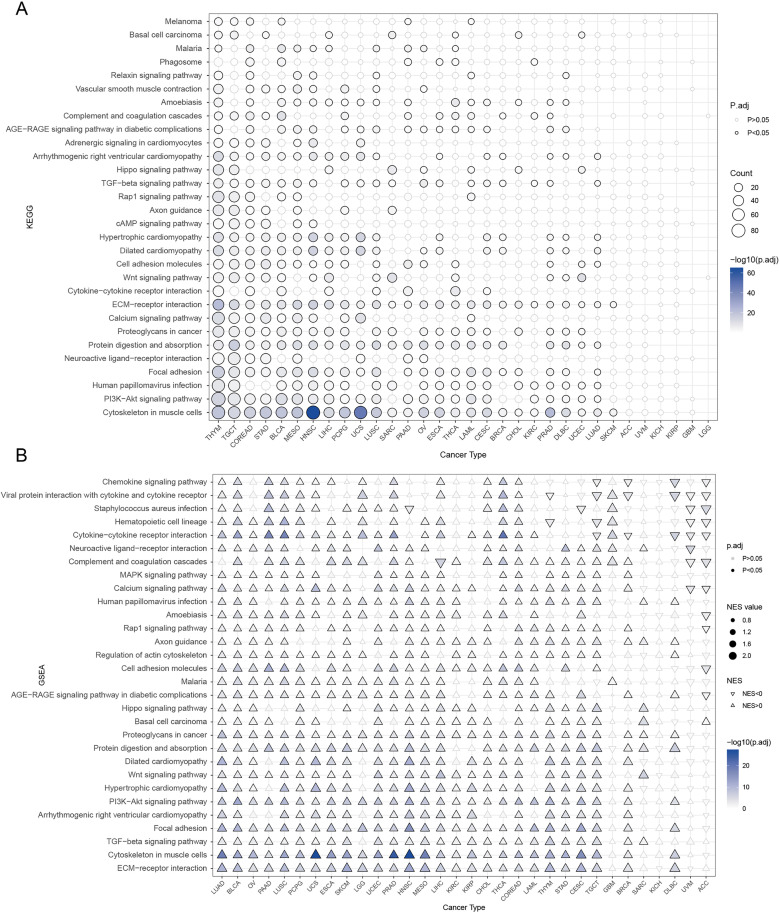
Enrichment Analysis of MATN3. **(A)** Dot plot representing the enrichment analysis of MATN3-associated pathways in various cancer types using the Kyoto Encyclopedia of Genes and Genomes (KEGG). **(B)** Triangle plot showing results from Gene Set Enrichment Analysis (GSEA) for MATN3 across different cancer types.

The KEGG analysis highlighted significant enrichment in the “Cytoskeleton in muscle cells” pathway across multiple cancer types. Additionally, there was substantial enrichment in pathways such as PI3K-Akt signaling, Focal adhesion, and ECM-receptor interaction, which are intimately associated with the processes of cancer metastasis and invasion (Fig 7A). These pathways suggest a mechanistic link between MATN3 expression and the cellular dynamics that facilitate tumor cell migration and adhesion. Further insights were gained through GSEA, where the ECM-receptor interaction pathway showed significant enrichment across nearly all examined cancers. Other pathways that were prominently enriched include the TGF-beta signaling pathway, Focal adhesion, PI3K-Akt signaling pathway, and Wnt signaling pathway (Fig 7B). These pathways are fundamentally involved in regulating cell proliferation, survival, and differentiation, highlighting the potential multifaceted role of MATN3 in tumor biology and cellular behavior.

**Fig 8:**
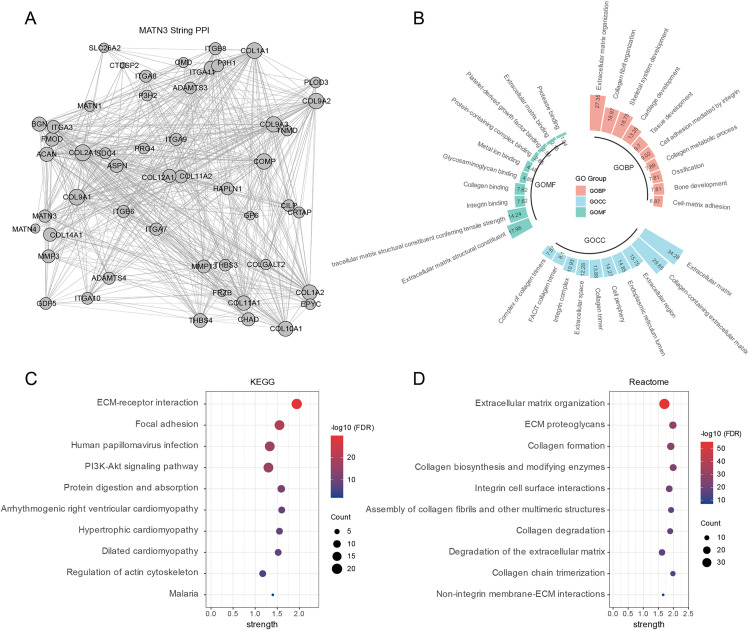
Enrichment Analysis of MATN3 Protein Interactions and Associated Pathways Using STRING Database. **(A)** Protein-Protein Interaction (PPI) network for MATN3 generated using the STRING database. **(B)** Gene Ontology (GO) enrichment analysis presented in a circular layout, subdivided into three main GO categories: Biological Process (BP), Cellular Component (CC), and Molecular Function (MF). Dot plot displaying the enrichment of **(C)** KEGG and **(D)** Reactome pathways associated with MATN3 based on the STRING database analysis.

In the final phase of our investigation into MATN3’s role in cancer, an interaction network was constructed using the STRING database, focusing on proteins related to MATN3 (Fig 8A). This protein-protein interaction (PPI) network provided a framework for further functional insights through Gene Ontology (GO) analysis. As depicted in Fig 8B, the GO analysis revealed that proteins associated with MATN3 are significantly involved in cellular functions related to extracellular mechanisms. This suggests a role for MATN3 in modulating the extracellular environment, which is crucial for various cellular processes including cell communication and migration.

Additionally, enrichment analyses were performed using both KEGG and REACTOME databases. These analyses reaffirmed the significant enrichment of pathways such as the PI3K-AKT signaling and ECM-related pathways. The recurrent identification of these pathways across different analytical platforms underscores their potential relevance in the context of MATN3 expression and activity in cancer, highlighting the integral role of these signaling cascades in modulating tumor behavior and interaction with the tumor microenvironment. As shown in Supplementary Figure 5A-B in S1 File, α-Pinene treatment led to a dose-dependent decrease in the phosphorylation levels of PI3K and AKT, while the total levels of PI3K and AKT slightly decreased. Additionally, MATN3 expression was reduced following α-Pinene exposure. These results support that α-Pinene suppresses the PI3K-AKT signaling pathway and downregulates MATN3 at the protein level, further substantiating our proposed mechanism.

## Discussion

Our investigation into the anticancer effects of α-Pinene on hepatocellular carcinoma cell line revealed its potent antiproliferative activity and ability to modulate gene expression. Interestingly, we observed a significant downregulation of MATN3, a gene previously associated with poor prognosis in various cancers. This finding prompted a comprehensive exploration of MATN3’s expression patterns, mutational landscape, and clinical implications across a spectrum of malignancies. Based on these results, and in line with emerging strategies in cancer therapy, it is worth considering the potential of combining natural compounds such as α-Pinene with drug repurposing approaches. For example, utilizing GLP-1 based therapies or proteasome-targeting agents alongside natural compounds with immunomodulatory effects may offer a promising prophylactic or therapeutic strategy, potentially enhance anticancer efficacy and improving patient outcomes [46,47].

The pan-cancer analysis unveiled a striking dichotomy in MATN3 expression, with consistent overexpression in numerous cancer types, including ACC, BLCA, BRCA, COAD, ESCA, GBM, LGG, HNSC, LIHC, PAAD, READ, STAD, and TGCT. This ubiquitous upregulation suggests a potential oncogenic role for MATN3 in these malignancies. Conversely, cancers like KICH, KIRP, and LUSC exhibited lower MATN3 expression, hinting at context-dependent functions or distinct regulatory mechanisms. The reduced expression in specific cancer types raises intriguing questions about MATN3’s biological roles and highlights the importance of a nuanced, cancer-specific approach when considering therapeutic targeting.

The clinical significance of MATN3 expression was further reinforced by our survival analyses, which revealed that higher levels of MATN3 were associated with shorter overall survival in patients with BLCA, CESC, HNSC, LGG, LIHC, MESO, OV, PCPG, and STAD. Additionally, Cox proportional hazards regression models confirmed MATN3’s prognostic value, identifying it as a predictor of worse disease-specific survival and progression-free intervals in several cancer types. These findings underscore the potential utility of MATN3 as a prognostic biomarker and highlight its pivotal role in cancer progression and metastasis.

Delving into the genetic landscape of MATN3, our mutational analyses revealed diverse patterns of copy number variations, amplifications, and mutations across different cancer types. While the overall mutation rate remains relatively low, specific mutations within critical domains like the vWFA domains could potentially impact MATN3’s functional interactions and contribute to its dysregulation in cancer. Furthermore, the correlation between MATN3 expression and indicators of tumor heterogeneity, such as tumor purity, tumor mutational burden, microsatellite instability, and homologous recombination deficiency, suggested a complex interplay between MATN3 and the tumor microenvironment.

Notably, our research has elucidated a significant positive correlation between MATN3 expression and cancer-associated fibroblasts, a pattern that is consistently observed across a majority of tumor types. CAFs are recognized as key components of the tumor microenvironment, playing a pivotal role in supporting tumor growth, invasion, and metastasis [48,49]. This relationship suggests a potential role for MATN3 in modulating the tumor stroma and influencing processes like angiogenesis and tumor-stromal crosstalk.

Furthermore, the positive correlation between MATN3 and various immunosuppressive genes, checkpoint inhibitors, and chemokines in several cancer types implies a potential involvement in immune evasion and regulation of the anti-tumor immune response. MATN3 shows a strong positive correlation with immune genes in cancers including LIHC, PAAD, COADREAD, and PRAD. Its association with negative prognostic factors in these tumor types highlights its potential importance in immune regulation and disease outcome. Given its impact on prognosis, further detailed research on MATN3 in these cancers is crucial for understanding its biological roles and therapeutic potential. These findings imply that MATN3 may shape the tumor immune microenvironment and influence the response to immune checkpoint blockade therapies. Further research is needed to determine whether MATN3 expression could serve as a predictive biomarker for immunotherapy or as a potential therapeutic target to improve patient outcomes.

The enrichment analyses shed light on the potential mechanisms underlying MATN3’s functions in cancer. Pathways such as cytoskeleton organization, PI3K-Akt signaling, focal adhesion, and ECM-receptor interaction were significantly enriched, suggesting that MATN3 may play a role in regulating cellular dynamics, migration, and invasion [50,51]. Additionally, the enrichment of pathways like TGF-beta signaling and Wnt signaling implicate MATN3 in processes related to cell proliferation, survival, and differentiation [52–54]. The protein-protein interaction network and Gene Ontology analyses further reinforced the potential involvement of MATN3 in extracellular mechanisms and cell communication, underscoring its multifaceted roles in the tumor microenvironment.

A key limitation of our study is the exclusive use of HepG2 and Hep3B cell lines for in vitro experiments. While these cell lines are widely used and represent important models of HCC, they may not capture the genetic and phenotypic heterogeneity observed in primary HCC. Future studies should include a broader range of HCC cell lines, as well as patient-derived organoids or xenograft models, to validate the generalizability of our findings and better reflect the complexity of hepatocellular carcinoma biology.

Our study combines in vitro experiments with comprehensive pan-cancer bioinformatics analysis, providing evidence for the role of MATN3 in cancer progression. The integration of molecular, cellular, and clinical data strengthens the translational relevance of our findings. The findings from this comprehensive analysis not only deepen our understanding of MATN3’s roles in tumorigenesis but also pave the way for future explorations into its potential as a prognostic biomarker, therapeutic target, and a window into the intricate interplay between cancer cells and their microenvironment. Unraveling the intricate mechanisms underlying MATN3’s functions could unveil novel therapeutic avenues and contribute to the development of personalized treatment strategies tailored to the unique molecular landscapes of different cancer types. Looking forward, the clinical translation of α-Pinene and MATN3-targeted therapies presents both opportunities and challenges. Further preclinical studies, including in vivo efficacy and toxicity assessments, are necessary to establish the safety and therapeutic potential of α-Pinene in HCC. Additionally, the development of specific MATN3 inhibitors or strategies to modulate MATN3 expression could provide novel options for targeted therapy. Integrating these approaches with existing treatment modalities, such as immune checkpoint inhibitors, may further enhance therapeutic outcomes. Ultimately, prospective clinical trials will be essential to evaluate the efficacy of these strategies in HCC patients.

## Supporting information

S1 File **S1 Fig.** The MATN3 mRNA expression after treatment with α-Pinene. **S2 Fig.** The expression of MATN3 in TCGA. **S3 Fig.** Forest plots depicting the association of MATN3 expression with disease-specific survival (DSS) and progression-free interval (PFI) across multiple cancer types. **S4 Fig.** Correlation between MATN3 expression and tumor heterogeneity indices across multiple cancer types. **S5_Fig.** Effects of α-Pinene on the expression of PI3K/AKT signaling pathway proteins and MATN3 in (A) Hep3B and (B) HepG2. **S1 Table.** The association of MATN3 expression with overall survival (OS) across multiple cancer types. **S2 Table.** Sequencing data.
(ZIP)

## Abbreviation:

ACC: Adrenocortical Carcinoma
AKT: AKT Serine/Threonine Kinase 1
ATCC: American Type Culture Collection
BCA: Bicinchoninic Acid
BLCA: Bladder Urothelial Carcinoma
BRCA: Breast Invasive Carcinoma
CAFs: Cancer-Associated Fibroblasts
CCLE: Cancer Cell Line Encyclopedia
CCK8: Cell Counting Kit-8
cDNA: Complementary DNA
CNV: Copy Number Variation
COAD: Colon Adenocarcinoma
COADREAD: Colon and Rectum Adenocarcinoma
CST: Cell Signaling Technology
DMSO: Dimethyl Sulfoxide
DSS: Disease-Specific Survival
ECM: Extracellular Matrix
EMEM: Eagle’s Minimum Essential Medium
EPIC: Estimating the Proportion of Immune and Cancer cells
ESCA: Esophageal Carcinoma
ESTIMATE: Estimation of STromal and Immune cells in MAlignant Tumor tissues using Expression data
FBS: Fetal Bovine Serum
GBM: Glioblastoma Multiforme
GAPDH: Glyceraldehyde-3-Phosphate Dehydrogenase
GO: Gene Ontology
GTEx: Genotype-Tissue Expression
GSEA: Gene Set Enrichment Analysis
HCC: Hepatocellular Carcinoma
HNSC: Head and Neck Squamous Cell Carcinoma
HRD: Homologous Recombination Deficiency
KEGG: Kyoto Encyclopedia of Genes and Genomes
KICH: Kidney Chromophobe
KIRP: Kidney Renal Papillary Cell Carcinoma
LGG: Lower Grade Glioma
LIHC: Liver Hepatocellular Carcinoma
LUSC: Lung Squamous Cell Carcinoma
MATN3: Matrilin-3
MESO: Mesothelioma
MSI: Microsatellite Instability
mRNA: Messenger RNA
NK: Natural Killer
NSCLC: Non-Small Cell Lung Cancer
OV: Ovarian Serous Cystadenocarcinoma
PAAD: Pancreatic Adenocarcinoma
PCPG: Pheochromocytoma and Paraganglioma
PFI: Progression-Free Interval
PI3K: Phosphoinositide 3-Kinase
PPI: Protein-Protein Interaction
PRAD: Prostate Adenocarcinoma
PVDF: Polyvinylidene Fluoride
qPCR: Quantitative Polymerase Chain Reaction
RIN: RNA Integrity Number
RNA: Ribonucleic Acid
RT-qPCR: Reverse Transcription Quantitative PCR
SD: Standard Deviation
SDS-PAGE: Sodium Dodecyl Sulfate Polyacrylamide Gel Electrophoresis
STAD: Stomach Adenocarcinoma
STRING: Search Tool for the Retrieval of Interacting Genes/Proteins
TCGA: The Cancer Genome Atlas
TGCT: Testicular Germ Cell Tumor
THCA: Thyroid Carcinoma
THYM: Thymoma
TMB: Tumor Mutational Burden
TNFSF4: Tumor Necrosis Factor Ligand Superfamily Member 4
vWFA: von Willebrand Factor A domain
WGBS: Whole Genome Bisulfite Sequencing
XENA: UCSC Xena Browser
