# Supplementary figures and images for: Regulatory influence of α-Pinene on MATN3 expression in hepatocellular carcinoma: Extending to pan-cancer analysis
Source: PLoS One. 2025 Sep 26;20(9):e0330653. doi: 10.1371/journal.pone.0330653 (PMC12468984; doi:10.1371/journal.pone.0330653)

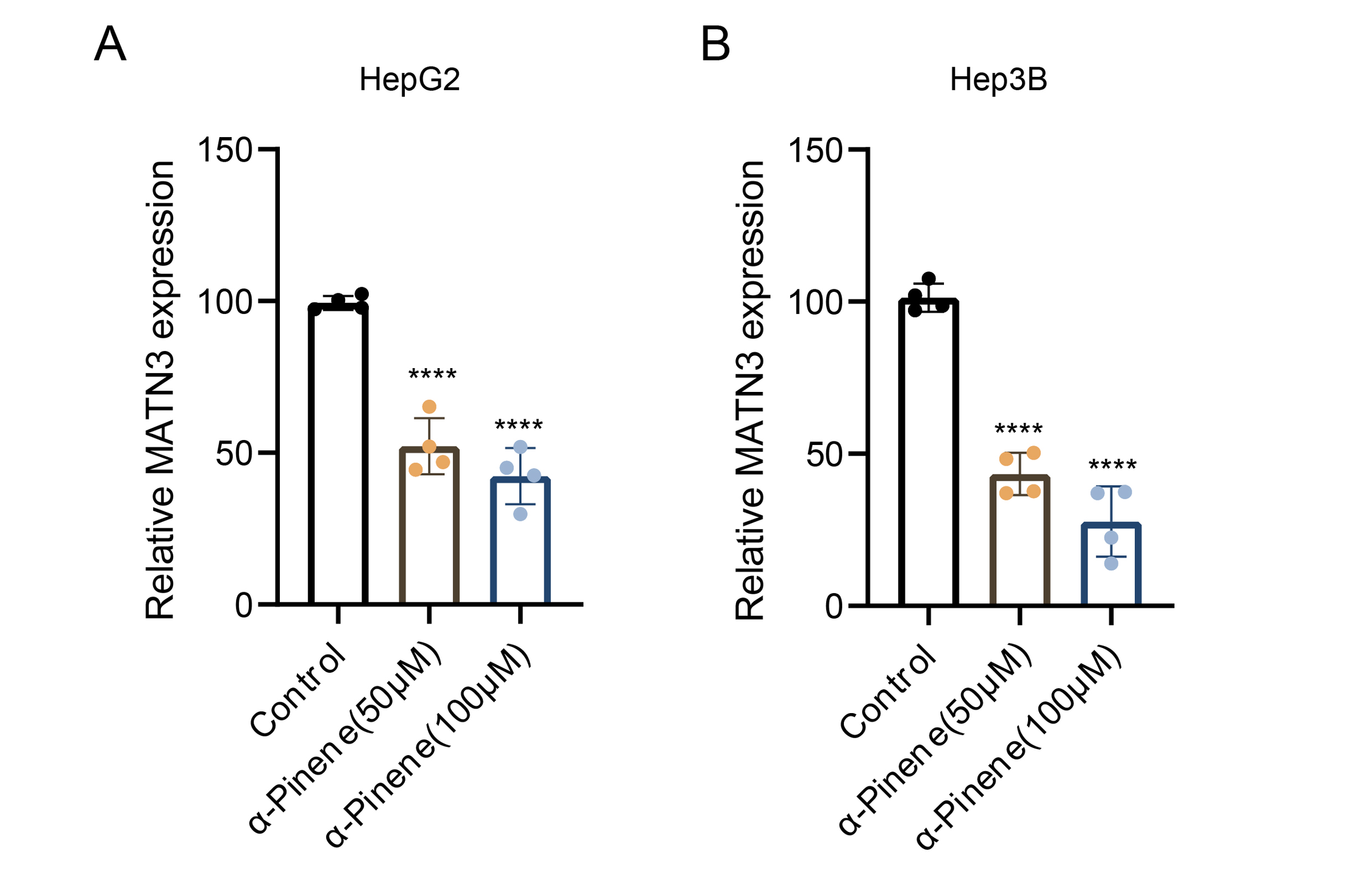

Supplement: S1 File — S1 Fig. The MATN3 mRNA expression after treatment with α-Pinene. S2 Fig. The expression of MATN3 in TCGA. S3 Fig. Forest plots depicting the association of MATN3 expression with disease-specific survival (DSS) and progression-free interval (PFI) across multiple cancer types. S4 Fig. Correlation between MATN3 expression and tumor heterogeneity indices across multiple cancer types. S5_Fig. Effects of α-Pinene on the expression of PI3K/AKT signaling pathway proteins and MATN3 in (A) Hep3B and (B) HepG2. S1 Table. The association of MATN3 expression with overall survival (OS) across multiple cancer types. S2 Table. Sequencing data. (ZIP) [file pone.0330653.s001.zip › S1_Fig.tif]

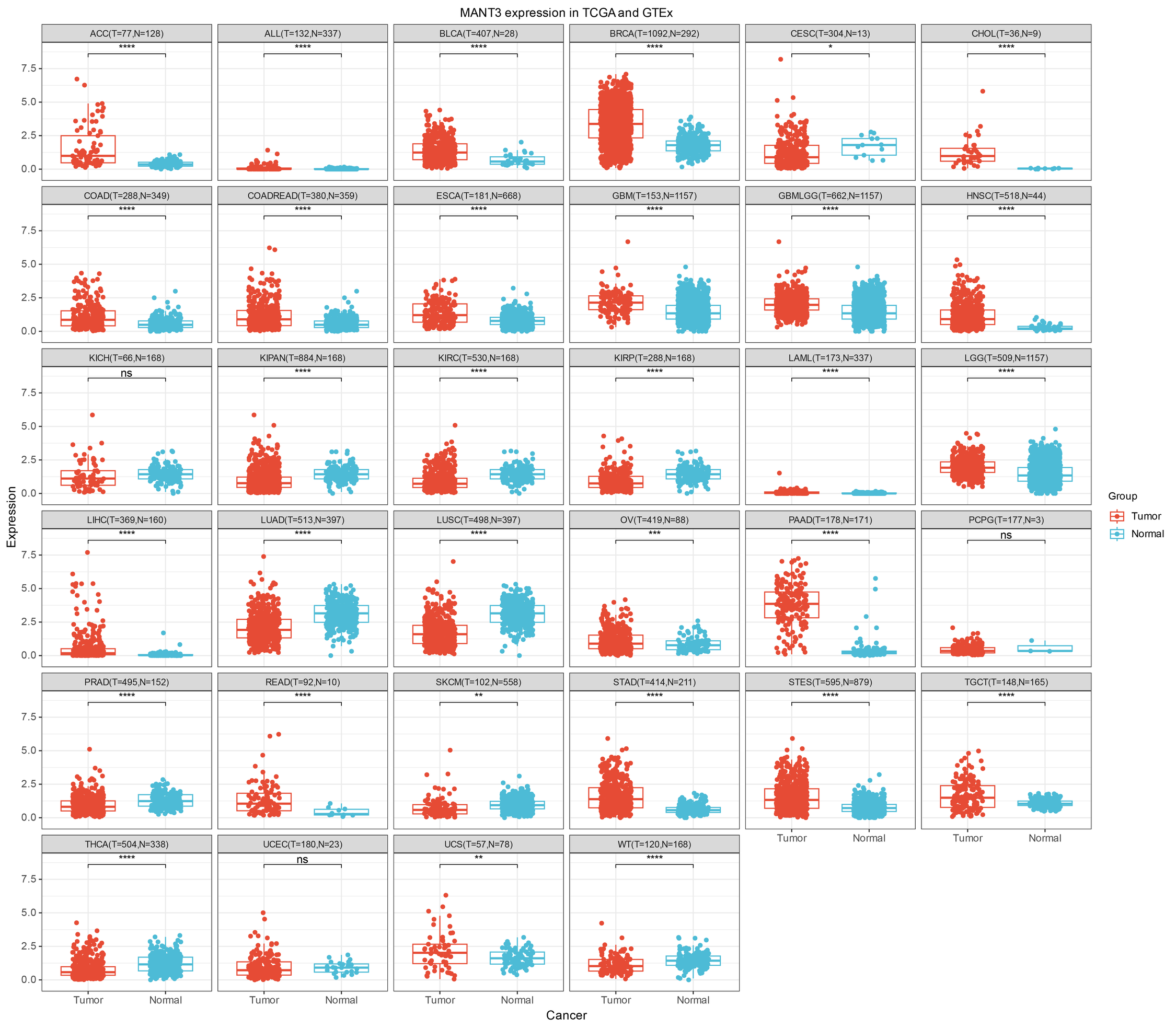

Supplement: S1 File — S1 Fig. The MATN3 mRNA expression after treatment with α-Pinene. S2 Fig. The expression of MATN3 in TCGA. S3 Fig. Forest plots depicting the association of MATN3 expression with disease-specific survival (DSS) and progression-free interval (PFI) across multiple cancer types. S4 Fig. Correlation between MATN3 expression and tumor heterogeneity indices across multiple cancer types. S5_Fig. Effects of α-Pinene on the expression of PI3K/AKT signaling pathway proteins and MATN3 in (A) Hep3B and (B) HepG2. S1 Table. The association of MATN3 expression with overall survival (OS) across multiple cancer types. S2 Table. Sequencing data. (ZIP) [file pone.0330653.s001.zip › S2_Fig.tif]

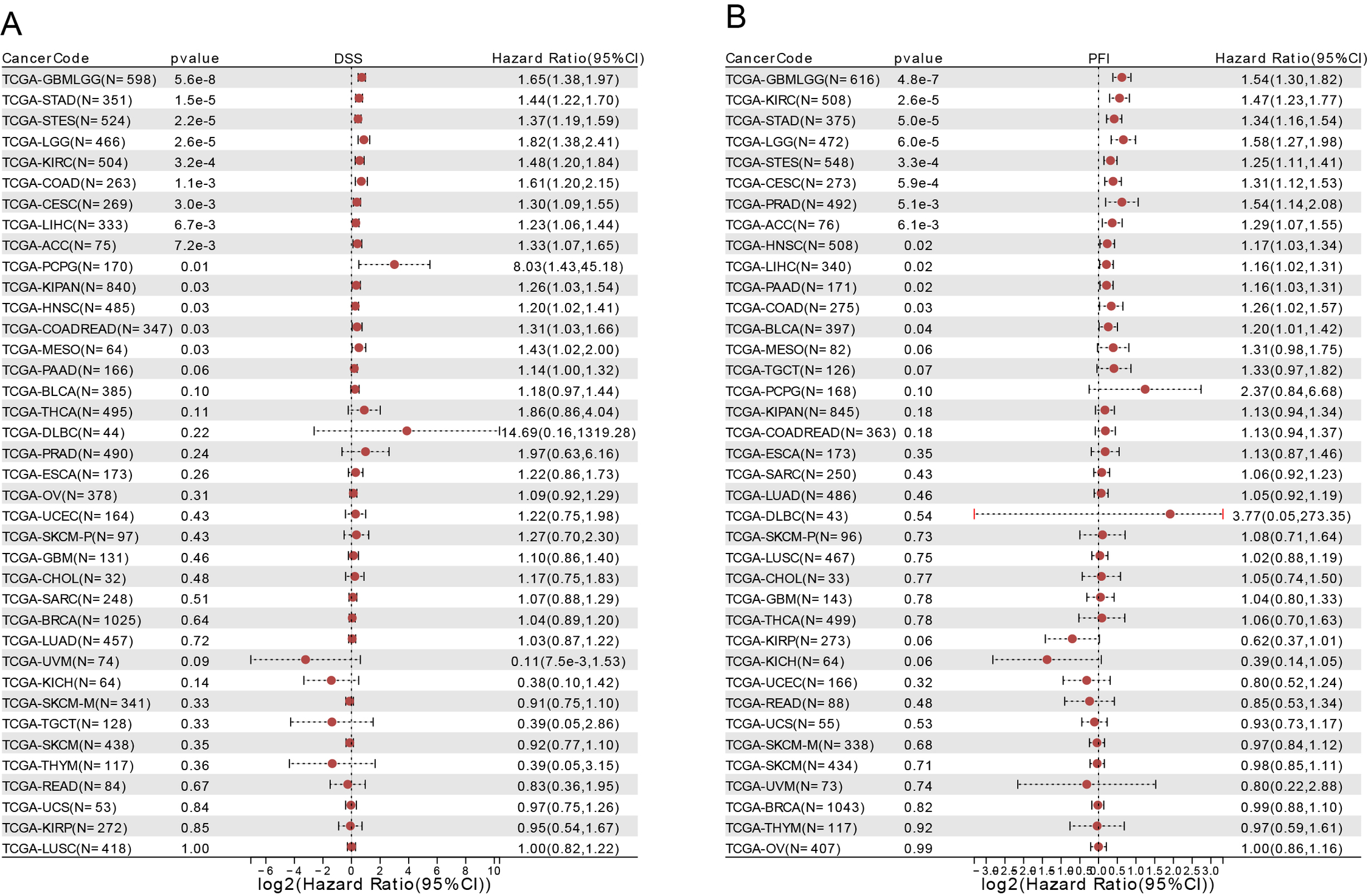

Supplement: S1 File — S1 Fig. The MATN3 mRNA expression after treatment with α-Pinene. S2 Fig. The expression of MATN3 in TCGA. S3 Fig. Forest plots depicting the association of MATN3 expression with disease-specific survival (DSS) and progression-free interval (PFI) across multiple cancer types. S4 Fig. Correlation between MATN3 expression and tumor heterogeneity indices across multiple cancer types. S5_Fig. Effects of α-Pinene on the expression of PI3K/AKT signaling pathway proteins and MATN3 in (A) Hep3B and (B) HepG2. S1 Table. The association of MATN3 expression with overall survival (OS) across multiple cancer types. S2 Table. Sequencing data. (ZIP) [file pone.0330653.s001.zip › S3_Fig.tif]

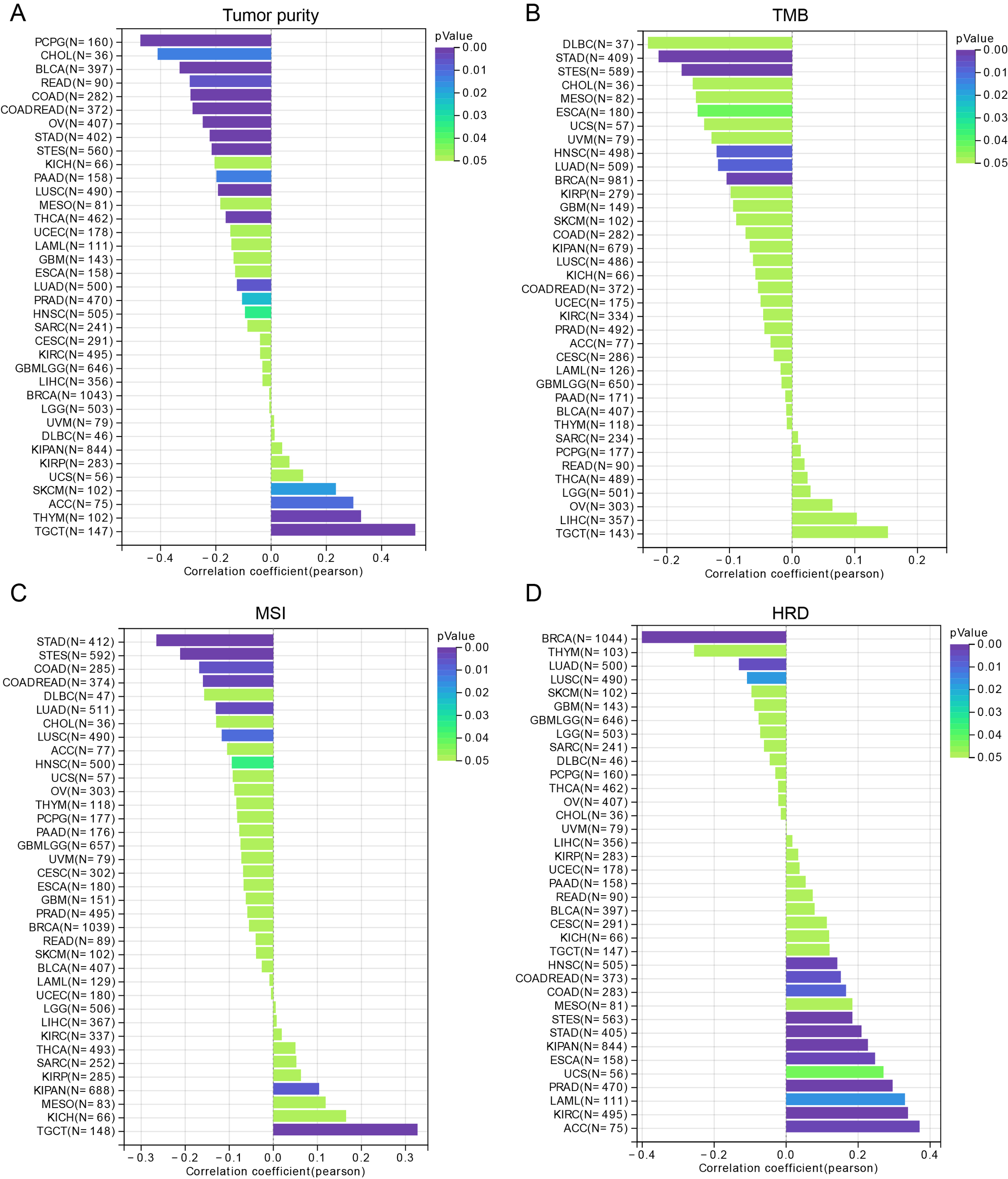

Supplement: S1 File — S1 Fig. The MATN3 mRNA expression after treatment with α-Pinene. S2 Fig. The expression of MATN3 in TCGA. S3 Fig. Forest plots depicting the association of MATN3 expression with disease-specific survival (DSS) and progression-free interval (PFI) across multiple cancer types. S4 Fig. Correlation between MATN3 expression and tumor heterogeneity indices across multiple cancer types. S5_Fig. Effects of α-Pinene on the expression of PI3K/AKT signaling pathway proteins and MATN3 in (A) Hep3B and (B) HepG2. S1 Table. The association of MATN3 expression with overall survival (OS) across multiple cancer types. S2 Table. Sequencing data. (ZIP) [file pone.0330653.s001.zip › S4_Fig.tif]

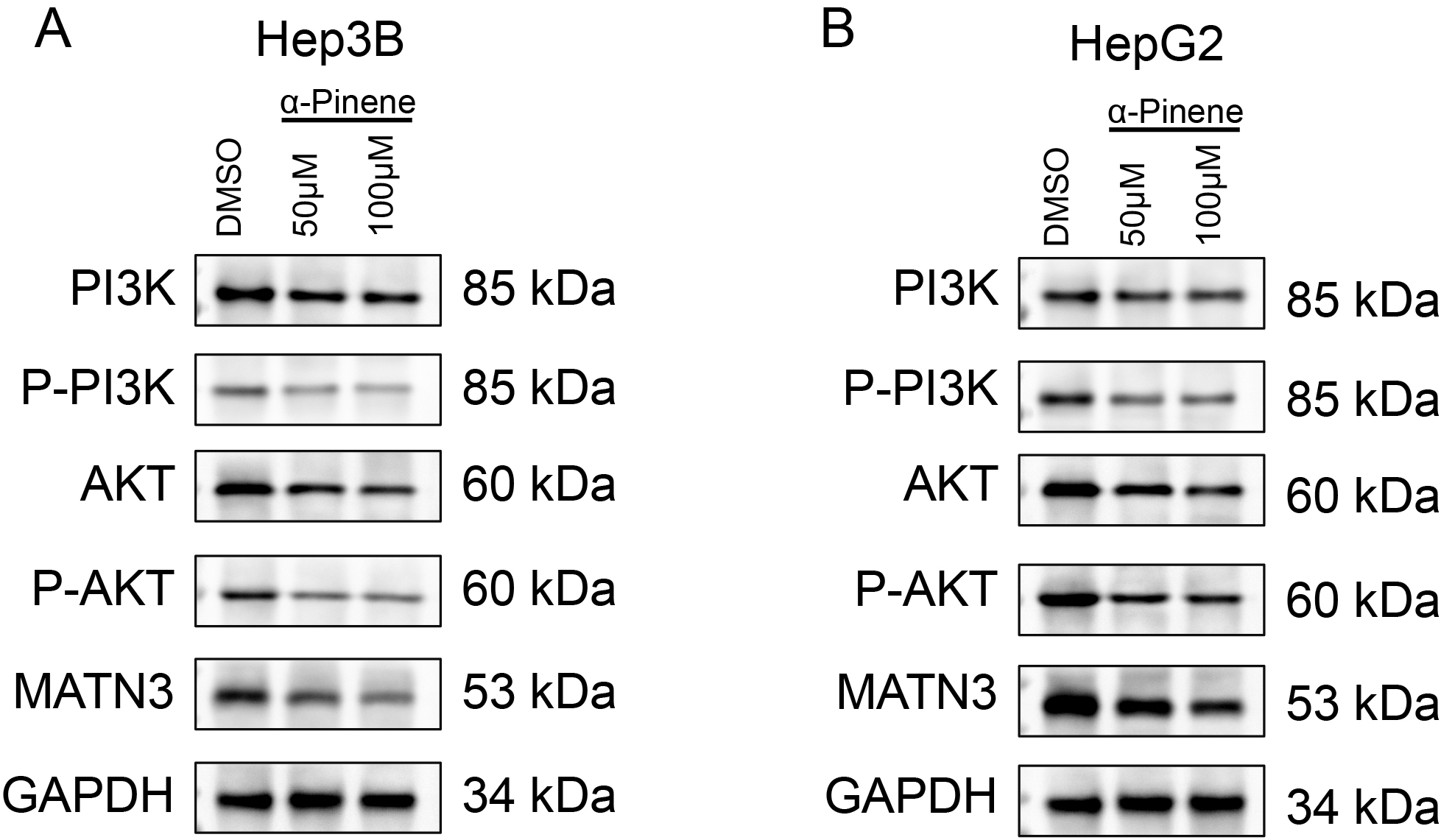

Supplement: S1 File — S1 Fig. The MATN3 mRNA expression after treatment with α-Pinene. S2 Fig. The expression of MATN3 in TCGA. S3 Fig. Forest plots depicting the association of MATN3 expression with disease-specific survival (DSS) and progression-free interval (PFI) across multiple cancer types. S4 Fig. Correlation between MATN3 expression and tumor heterogeneity indices across multiple cancer types. S5_Fig. Effects of α-Pinene on the expression of PI3K/AKT signaling pathway proteins and MATN3 in (A) Hep3B and (B) HepG2. S1 Table. The association of MATN3 expression with overall survival (OS) across multiple cancer types. S2 Table. Sequencing data. (ZIP) [file pone.0330653.s001.zip › S5_Fig.tif]
